# Sequence and expression pattern of the germ line marker *vasa* in honey bees and stingless bees

**DOI:** 10.1590/S1415-47572009005000043

**Published:** 2009-09-01

**Authors:** Érica Donato Tanaka, Klaus Hartfelder

**Affiliations:** Departamento de Biologia, Faculdade de Filosofia, Ciências e Letras de Ribeirão Preto, Universidade de São Paulo, Ribeirão Preto, SPBrazil; 2 Institut de Biologia Evolutiva, Consejo Superior de Investigaciones Científicas, Universitat Pompeu Fabra, BarcelonaSpain; 3 Departamento de Biologia Celular e Molecular e Bioagentes Patogênicos, Faculdade de Medicina de Ribeirão Preto, Universidade de São Paulo, Ribeirão Preto, SPBrazil

**Keywords:** *Apis mellifera*, *Melipona quadrifasciata*, *vasa*, social insect, oogenesis

## Abstract

Queens and workers of social insects differ in the rates of egg laying. Using genomic information we determined the sequence of *vasa*, a highly conserved gene specific to the germ line of metazoans, for the honey bee and four stingless bees. The *vasa* sequence of social bees differed from that of other insects in two motifs. By RT-PCR we confirmed the germ line specificity of *Amvasa* expression in honey bees. *In situ* hybridization on ovarioles showed that *Amvasa* is expressed throughout the germarium, except for the transition zone beneath the terminal filament. A diffuse *vasa* signal was also seen in terminal filaments suggesting the presence of germ line cells. Oocytes showed elevated levels of *Amvasa* transcripts in the lower germarium and after follicles became segregated. In previtellogenic follicles, *Amvasa* transcription was detected in the trophocytes, which appear to supply its mRNA to the growing oocyte. A similar picture was obtained for ovarioles of the stingless bee *Melipona quadrifasciata*, except that *Amvasa* expression was higher in the oocytes of previtellogenic follicles. The social bees differ in this respect from *Drosophila*, the model system for insect oogenesis, suggesting that changes in the sequence and expression pattern of *vasa* may have occurred during social evolution.

## Introduction

The decision of who is to reproduce and who is not is a key question in the evolution of sociality. In the highly eusocial Hymenoptera, this decision has become fixed into a system of reproductive division of labor between two morphologically distinct female phenotypes, the queen and worker. Honey bees (Apini) and stingless bees (Meliponini) belong to the subfamily Apinae (Michener, 2000). Both are highly eusocial, but they differ considerably in their life history characteristics (Sakagami, 1982; Imperatriz-Fonseca and Kleinert, 1998). Besides exhibiting a much more flexible age polyethism (Waldschmidt and Campos, 1997) than *Apis mellifera,* a major difference between stingless bees and honey bees concerns ovary development and worker reproduction (Hartfelder *et al.*, 2006).

When a honey bee queen emerges from the brood cell, each of her two ovaries consists of 180-200 ovarioles. These are activated soon after mating, allowing her to lay up to 2,000 eggs per day. In contrast, adult workers have only 2-10 ovarioles in each ovary (Snodgrass, 1956). In the presence of the queen, only very few workers show signs of progressive oogenesis (Visscher and Dukas, 1995), an exception being a mutant phenotype, the so-called anarchistic workers (Barron and Oldroyd, 2001).

Queens and workers of stingless bees do not exhibit such drastic differences in ovariole number and, in terms of gonad size, the two castes differ primarily in the length of the ovariole filaments (Hartfelder and Engels, 1992; Cruz-Landim *et al.*, 1998). Interestingly, worker reproduction in stingless bees covers the entire spectrum, from full fertility in the presence of the queen (Imperatriz-Fonseca and Kleinert, 1998) to complete sterility due to ovary degeneration during metamorphosis (Boleli *et al.*, 1999). This marked variation in ovary structure and reproductive biology makes social bees an interesting group for comparative studies on oogenesis.

Recent studies on oogenesis progress and control in *Apis mellifera* have raised several questions concerning the presence of putative germ-line stem cells in the terminal filament (Gutzeit *et al.*, 1993; Tanaka and Hartfelder, 2004), on programmed cell death during oogenesis (Tanaka *et al.*, 2006), and on the control of vitellogenesis via expression of the vitellogenin receptor encoding gene (Guidugli-Lazzarini *et al.*, 2008). These studies generally compared honey bee oogenesis with corresponding processes in the fruit fly, *Drosophila melanogaster*, which also has a polytrophic meroistic type of ovary and for which the genetics of germ cell determination and differentiation are much better understood (for reviews see, King, 1970; de Cuevas *et al.*, 1997; Gilboa and Lehmann, 2004).

Of specific interest to the determination of the germ line is the *vasa* gene product. Vasa protein, an ATP-dependent DEAD-box RNA helicase (Hay *et al.*, 1988; Pyle, 2008), is a conserved germ line-specific marker for most metazoan phyla (Fujiwara *et al.*, 1994; Gruidl *et al.*, 1996; Olsen *et al.*, 1997; Shibata *et al.*, 1999; Mochizuki *et al.*, 2001; Extavour and Akam, 2003; Sagawa *et al.*, 2005; Rebscher *et al.*, 2007). In *Drosophila* oogenesis, Vasa protein surrounds germ cell nuclei in the germarium and in early egg chambers. Later on (stage 10), *vasa* mRNA appears at first homogeneously distributed throughout the oocyte cytoplasm before the protein becomes asymmetrically segregated to the posterior pole (Hay *et al.*, 1988; Liang *et al.*, 1994; Sano *et al.*, 2002; Findley *et al.*, 2003). In the grasshopper *Schistocerca gregaria,* which has a panoistic type ovary, *vasa* mRNA, as well as Vasa protein are highly expressed in the early stages of oogenesis, with levels rapidly falling in maturing oocytes (Chang *et al.*, 2002).

Due to the highly conserved association of *vasa* expression with germ line development and gametogenesis in metazoans (Extavour, 2007), efforts were made to identify a *bona fidevasa* ortholog in the sequenced honey bee genome (Dearden *et al.*, 2006; The Honey Bee Genome Sequencing Consortium, 2006). Since this computationally predicted *vasa* sequence revealed a divergence in one of the characteristic Vasa motifs, being a change from ARKF to IVKF (Dearden *et al.*, 2006), the question became whether this change may be specific to the honey bee, as part of its special social biology, or whether it may have preceded social evolution in the corbiculate bees. Furthermore, we were interested to see whether *vasa* gene function, and especially its localization in the germ cells may differ between honey bees and stingless bees, as part of their divergence in worker reproduction. In the present study we sequenced *vasa* orthologs for four species of stingless bees and, by RT-PCR and *in situ* hybridization with a honey bee *vasa* probe, we compared *vasa* expression patterns in *Apis mellifera* ovaries with those of the stingless bee *Melipona quadrifasciata*.

## Materials and Methods

###  Bees

*Apis mellifera* queens and workers were obtained from Africanized honey bee stocks. Worker larvae and pupae were collected directly from brood frames and staged (Michelette and Soares, 1993). Queens were reared following standard apicultural procedures. Pharate adult queens and workers were allowed to hatch in an incubator (34 °C, 80% r.h.). Newly emerged workers were paint-marked and reintroduced into queenless hives, from where they were retrieved two weeks later when their ovaries were expected to be active (Makert *et al.*, 2006). Queens that were to be aged without mating (virgin queens) were caged individually in plastic queen cages and were kept in hives until they reached the appropriate age. Other queens were paint-marked after hatching and were shortly thereafter introduced into queenless hives where they were allowed to mate naturally. Once they started to lay eggs they were retrieved for ovary analysis.

*Melipona quadrifasciata* queens and workers were collected from laboratory colonies. To obtain virgin queens of known age, combs with pharate-adult brood were removed and transferred to an incubator (28 °C, 80% r.h.) for up to 16 h. Newly emerged queens were kept in Petri dishes, each accompanied by five workers, and were maintained in the incubator until they reached the adequate age for analysis. These bees received sugar water, fermented pollen paste and water *ad libitum*. Specimens of the other three stingless bee species, *Melipona scutellaris, Scaptotrigona postica* [frequently also referred to as *Scaptotrigonaaff. depilis,* due to uncertainties in the taxonomy of this genus, (Camargo and Pedro, 2007)] and *Frieseomelitta varia* were also collected from brood frames of laboratory colonies, as described for *M. quadrifasciata.*

###  Sequencing and bioinformatics analysis

For experimental confirmation of the computationally predicted honey bee *vasa* ortholog (GB14804-PA) (Dearden, 2006; The Honey Bee Genome Sequencing Consortium, 2006) we designed gene-specific primers aligning to five regions in the predicted coding sequence (V7F0: 5'-ATGGCTGATGACTGGGGT-3'; V7R1: 5'-CCATA ACTACGTCCACCTTC-3'; V7F2: 5'-GAGGAAAGTT GTCTGCTGG-3'; V7R2: 5'-CCAGCAGACAACTTTC CTC-3'; V7F3: 5'-GCCGTTTTCTTATCCGAG-3'; V7R3: 5'-CTCGGATAAGAAAACGGC-3'; V7R4: 5'-CCGGTTCTT TGCTACG-3') and one in the predicted 3'UTR region (V7R5: 5'-GAAACAAAGCTTACTACC CTG-3'). RT-PCR conditions were as follows: 95 °C for 5 min, followed by 35 cycles of 94 °C for 30 s, 50 °C for 30 s, 72 °C for 30 s, and a final extension step at 72 °C for 7 min. The amplification products of expected size (V7F0+V7R1: 100 bp, V7F0+V7R2: 900 bp, V7F2+V7R3: 500 bp, V7F2+V7R4: 900 bp, V7F3+V7R4: 500 bp, V7F3+V7R5: 700 bp) were ligated into pGEM-T *Easy* vector (Promega) for transformation of competent DH5α*E. coli* cells.

To identify *vasa* orthologs in the four stingless bee species, *Melipona quadrifasciata, Melipona scutellaris, Frieseomelitta varia and Scaptotrigona postica,* we used primer combinations originally designed to amplify *Apis mellifera vasa* (V7F0+V7R2, V7F2+V7R3, V7F3+V7R4). DNA sequencing was performed by the dideoxy sequencing method, using a BigDye terminator v3.0 Cycle Sequencing Ready Reaction (Applied Biosystems) in an ABI Prism 310 Genetic Analyzer (Applied Biosystems).

For annotation of the honey bee *vasa* gene, the CDS assembled from the RT-PCR products was aligned against the genome sequence (Amel. 4.0, The Honey Bee Genome Sequencing Consortium, 2006) using a LINUX-based Artemis platform (version 7.1, The Sanger Institute). Sequence homology searches were performed by BLAST (blastx algorithm) to retrieve putative *vasa* orthologs of two other hymenopteran species, *Copidosoma floridanum* (GenBank accession number AAT11555.1) and *Nasonia vitripennis* (GenBank accession number XM_001603906.1). The *vasa* orthologs of these two species and those of the honey bee and the four stingless bee species obtained in this study were used in ClustalW multiple alignments, with *D. melanogaster vasa* as an outgroup. The ClustalW results were transformed to Mega 3.1 format (Kumar *et al.*, 2004). The Neighbor joining procedure (Saitou and Nei, 1987) was used for tree construction, which was evaluated by 1000 bootstrap repetitions.

###  RT-PCR analysis of *vasa* expression in *Apis mellifera* ovaries

RNA was extracted using TRIzol reagent (Invitrogen) from pools of 15-20 ovary pairs each. First-strand cDNA was synthesized using an oligo(dT)_12-18_ primer (Invitrogen) and Superscript II (Invitrogen) reverse transcriptase. RT-PCR reactions were carried out using the *Amvasa* primers V7F2 and V7R3 at 94 °C for 2 min followed by 32 cycles of 94 °C for 30 s, 55 °C for 30 s, 72 °C for 30 s, and a final extension step at 72 °C for 7 min. For normalization we amplified a cytoplasmatic actin gene fragment (GenBank accession number AB023025), an endogenous control gene appropriate for quantitative gene expression analysis in honey bees (Lourenço *et al.*, 2008). The number of amplification cycles was adjusted to avoid saturation. The *Amvasa* amplification products were analyzed by electrophoresis in 1% agarose gels containing ethidium bromide.

For the analysis of *vasa* expression in honey bee tissues other than the ovaries we prepared first-strand cDNA from brain, fat body, midgut, thorax (dorsal tegument) and abdomen (dorsal tegument) from newly emerged queens and workers. Additional samples were prepared from spermathecae of queens and from hypopharyngeal glands of workers, both of which are organs of caste-specific functions.

### *In situ* hybridization

For *in situ* localization of *vasa* transcripts in honey bee and stingless bee ovaries, sense and antisense probes were synthesized using *Amvasa-*specific primers containing a T7 promoter sequence (underlined) at the respective 5'-ends (ISH-F1: 5'-TAATACGACTCACTATAGGGCG AGGTAGAGGTCATGGTAAAGGAG-3' and ISH-R1: 5'-TAATACGACTCACTATAGGGCGAGAGGCACATTATCTCCACTCAC-3'), in combination with corresponding primers lacking the T7 sequence (ISH-F2 and ISH-R2). Amplification parameters were: 94 °C for 2 min, followed by 40 cycles of 94 °C for 40 s, 57 °C for 40 s, 72 °C for 40 s, and a final extension step at 72 °C for 7 min. PCR reactions with these primers generated a product of 331 bp. The antisense and sense products were produced by the primer combinations ISH-F2 + ISH-R1 and ISH-F1+ ISH-R2, respectively.

Aliquots of the amplification products were checked on agarose gels, purified (Wizard® SV Gel and PCR Clean-Up System, Promega) and quantified spectrophotometrically. RNA probes were generated by *in vitro* transcription from the T7 promoter using the DIG RNA Labelling Kit (SP6/T7) (Roche Applied Science). The transcription products were precipitated by addition of 1 μL ammonium acetate (10M) and 20 μL isopropanol. After centrifugation, the pellet was washed in 70% ethanol. The ethanol-free probes were resuspended in 50 μL hybridization buffer (50% formamide, 4 X SSC, 1 X Denhardt's solution, 250 μg/mL yeast extract, 250 μg/mL salmon sperm DNA, 50 μg/mL de heparin, 0,1% Tween-20, 5% dextrane sulfate) before storage at -20 °C.

Ovariole whole mounts were prepared for *A. mellifera* workers kept in queenright and queenless conditions, for mated and unmated queens, as well as for physogastric and virgin queens of *M. quadrifasciata*. After dissection of the ovaries, the ovarioles were separated in honey bee tissue culture medium (Rachinsky and Hartfelder, 1998) and the ovariole sheath was removed from each ovariole by means of watchmaker's forceps (number 5, Dumont). Fixation of the ovarioles and the subsequent hybridization and detection reactions were performed following the protocol optimized by Osborne and Dearden (2005) for *in situ* hybridization studies on honey bees.

## Results

###  Sequence characteristics of *Apis mellifera* and *Melipona quadrifasciata vasa* genes

**Figure 1:**
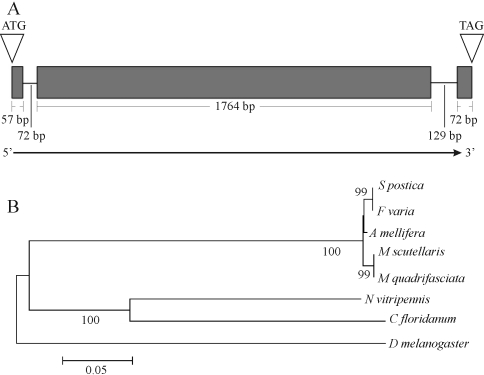
The *vasa* genes of highly eusocial bees. (A) Schematic diagram of the genomic region encoding the *vasa* gene of the honey bee *Apis mellifera*. The open arrowheads on the left and right indicate the start and stop codons, respectively. Exons are represented as boxes and their respective sizes are shown. Intron length was scaled to provide a general estimate of *vasa* gene size. (B) Molecular phylogeny for hymenopteran Vasa protein sequences. Vasa proteins of two species of parasitic wasps, *Nasonia vitripennis* and *Copidosoma floridanum* were aligned with Vasa protein sequences of the stingless bees *Melipona scutellaris*, *Melipona quadrifasciata, Scaptotrigona postica* and *Frieseomelitta varia*, as well as with *Apis mellifera* Vasa protein. *Drosophila melanogaster* Vasa was included as outgroup. ClustalW alignment results (Supplementary Material Figure S2) were used as input for tree construction by Neighbor joining (NJ). Results of 1,000 bootstrap repetitions are shown adjacent to the respective branches.

The *Apis mellifera vasa* gene spans an Open Reading Frame (ORF) of 1,893 bp, divided into 3 exons that are separated by 2 small introns (Figure 1A). The product sequenced in our study from overlapping RT-PCR fragments aligned 100% with the computationally predicted *Apis mellifera vasa* gene sequence (GenBank accession number ABC41341). Conceptual translation resulted in a predicted protein of 630 amino acids (AmVasa). Sequence similarity scores obtained by BLASTP analysis showed that AmVasa was 55 to 61% identical to the Vasa proteins of members of other insect orders, including the orthopterans *Schistocerca gregaria* and *Gryllus bimaculatus,* the lepidopteran *Bombyx mori*, the coleopteran *Tribolium castaneum,* and the dipterans *Aedes aegypti* , *Anopheles gambiae, Culex pipiens quinquefasciatus* and *Drosophila melanogaster* (Supplementary Material, Figure S1).

*Apis mellifera* Vasa contains all the nine conserved domains characteristic for the DEAD-box helicase family (GxxxPxxIQ, AxTGxGKT, PTRELA, TPGR, DEAD, SAT, GG, ARGLD, HRIGRTGR) and two of the conserved domains of the Vasa subfamily (GIVGxA, ExEExW) (Supplementary Material, Figure S1). AmVasa, however, differs in two potentially important aspects from the Vasa proteins of other insect species. First, the Vasa-specific ARKF domain presented a change in the first two amino acids, being IVKF in AmVasa. Second, the arginine-glycine-glycine (RGG) repeat, which is frequently found in N-terminal regions of many Vasa-related proteins, was represented in AmVasa by a single RGG copy only. The computationally predicted CDS encoding AmVasa (Dearden, 2006) was, thus, fully confirmed by assembly of RT-PCR fragments, including the modifications in the two above mentioned motifs.

For the stingless bee, *Melipona quadrifasciata,* conceptual translation of the *vasa* ortholog (partial cds, GenBank accession number FJ161962) resulted in a predicted protein (MqVasa) of 624 amino acid residues showing 98% sequence identity to *Apis mellifera* Vasa protein*.* A similarly high degree of identity as that between the *vasa* genes of *A. mellifera* and *M. quadrifasciata* was also obtained for the *vasa* orthologs of the three other stingless bee species, *Melipona scutellaris* (GenBank accession number EF601038), *Scaptotrigona postica* (GenBank accession number EF601037) and *Frieseomelitta varia* (GenBank accession number FJ161963). In the molecular phylogeny tree constructed by Neighbor Joining (Figure 1B) the Vasa sequences of the five bee species came out as a well supported branch, separated from the parasitic wasps *Nasonia vitripennis* and *Copidosoma floridanum*, both belonging to Chalcidoidea. Interestingly, the two *Melipona* species were also well separated from the two trigonine species *S. postica* and *F. varia.*

In the ClustalW alignments of the hymenopteran Vasa proteins, with *Drosophila* as outgroup (Supplementary Material - Figure S2), the following observations concerning modifications in characteristic Vasa motifs are noteworthy: (1) in the GxxxPxxIQ domain the five bee species are all GYKKPTPVQK, differing from *Nasonia* and *Copidosoma* in the third and fourth position, (2) in the PTRELA domain, *Copidosoma* is identical to *Drosophila*, differing from *Nasonia* and the bees, which are all PTRELT, (3) in the GIVGxA domain, *Nasonia* and *Copidosoma* are GIVGSA, whereas the bees and *Drosophila* are GIVGGA.

### *Amvasa* expression in the postembryonic gonad and in adult somatic tissues

**Figure 2:**
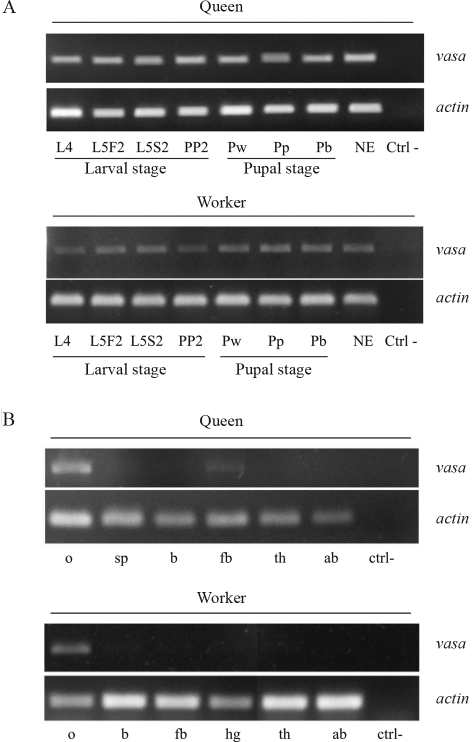
RT-PCR detection of *vasa* expression in honey bees. (A) Expression profile in ovaries of honey bee queens and workers during postembryonic development. L4 - 4^th^ larval instar; L5F2 - 5^th^ larval instar, mid-feeding phase; L5S2 - 5^th^ larval instar, mid-spinning phase; PP2 - mid-prepupal phase; Pw, Pp, Pb - white, pink and brown-eyed pupae, respectively; NE - newly emerged adult. Ctrl- - negative control, amplification without template. (B) Analysis of *vasa* expression in somatic tissues of adult queens and workers: o - ovary; b - brain; fb - fat body; th - thorax (dorsal tegument); ab - abdomen (dorsal tegument); sp - spermatheca of queen; hg - hypopharyngeal gland of worker. ctrl- - negative control without template. actin - *A. mellifera actin*, used for normalization.

Using specific primers we confirmed the presence of *Amvasa* transcripts in the ovaries of both castes of the honey bee throughout larval, pupal and imaginal development. Judging from band intensity, normalized against *actin* levels in the respective cDNA samples, the expression levels of *vasa* in ovaries of workers appear to be lower than in queens (Figure 2A). This observation is consistent with the differing dynamics of ovary development for the two castes (Hartfelder and Steinbrück, 1997). *Amvasa* transcript levels in the two castes showed little apparent modulation, suggesting that the gene is continuously expressed during postembryonic development of the gonads.

We next investigated whether *Amvasa* expression is gonad-specific. This analysis was conducted using RNA extracts from different body parts and organs of adult honey bee queens and workers. Apart from the expected expression in ovaries we could detect a faint but nevertheless clear *Amvasa* signal in dorsal fat body of queens (Figure 2B). Cloning and sequencing of this fragment confirmed that it was a *bona fidevasa* signal. We did not detect corresponding transcripts in fat body of workers, indicating possible caste-specific regulation of *Amvasa* expression in this key metabolic tissue. For all other tissues tested in this study there was no evidence for *Amvasa* expression, underlining its possible role in germ line specification and gonadal activity.

### *In situ* detection of *Amvasa* transcripts in ovaries of honey bee queens and workers

**Figure 3:**
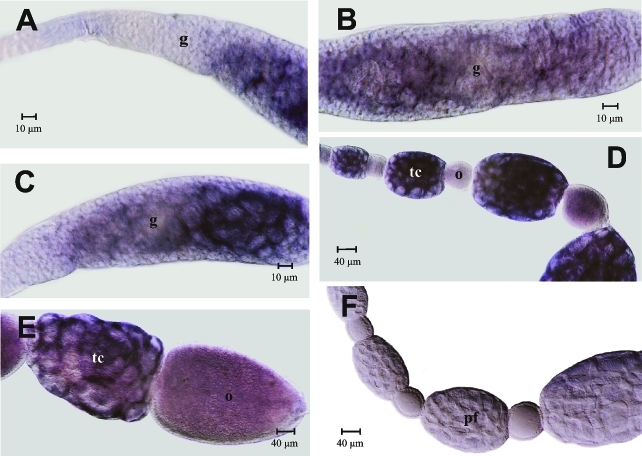
*Amvasa* mRNA detection by *in situ* hybridization in ovarioles of queenless honey bee workers. Apical is to the left in all figures. (A, B and C) *Amvasa* expression visualized in germ line cells throughout the germarium. The *Amvasa*-negative area at the left of A and C is the transition zone from the terminal filament to the germarium. (D) *Amvasa* expression in previtellogenic follicles is prominent in the trophic chambers and only gradually increases in the growing oocytes. (E) In early vitellogenic follicles, *Amvasa* transcripts in the oocyte show a homogeneous distribution. Negative control with sense probe (F). g - germarium, o - oocyte, pf - previtellogenic follicle, tc - trophic chamber, tf - terminal filament.

**Figure 4:**
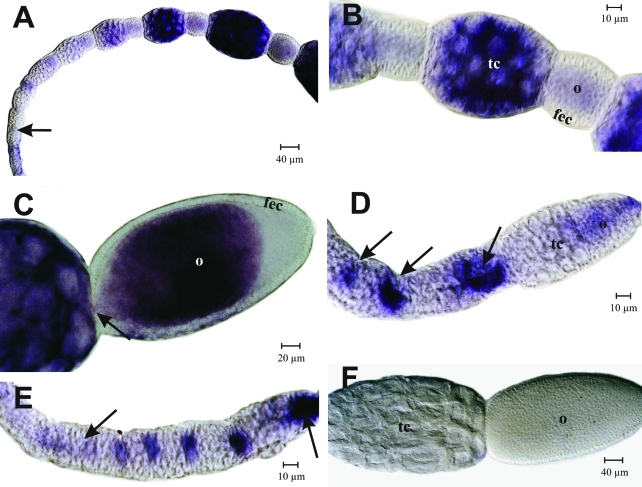
*Amvasa* mRNA detection by *in situ* hybridization in ovarioles of mated (A, B, C) and virgin queens (D, E) of *A. mellifera.* Shortly after follicles segregated from the germarium, *vasa* transcripts were visible primarily in oocytes (A, D, E, arrows). As the previtellogenic follicles matured, *Amvasa* expression was switched on and strongly increased in the trophocytes (A, B). In these follicles, *Amvasa* mRNA appeared to be transported into the oocyte across the trophocytic canal (C, arrow), gradually dispersing in the oocyte cytoplasm. (F) Negative control with sense probe. fec - follicular epithelium cells, o - oocyte, tc - trophic chamber.

The localization of *Amvasa* transcripts was investigated by *in situ* hybridization using a digoxigenin-labeled riboprobe. The general spatial pattern of *Amvasa* expression was very similar for the two honey bee castes (Figures 3  and 4) and was little influenced by social condition. We compared ovaries from queens of different social status (virgin queens and mated, egg laying queens) and from workers kept under queenless conditions, remembering that ovary activation in honey bee workers only exceptionally occurs in the presence of the queen (Hartfelder and Engels, 1998; Barron and Oldroyd, 2001; Makert *et al.*, 2006).

In ovaries of queenless workers, the onset of *Amvasa* expression was detected in the germarium (Figure 3A, B) at a position where early germ cell clusters consisting of 8-16 cells are formed (Tanaka and Hartfelder, 2004; Tanaka *et al.*, 2006). This point is just below the transition zone from the terminal filament to the germarium, a zone where we could not detect *Amvasa* transcripts (Figure 3A, C). Beneath this transition zone, expression levels gradually appeared to increase in germ cells (Figure 3C). After separation of the follicles from the germarium, *Amvasa* transcripts were primarily detected in nurse cells (trophocytes) (Figure 3D). In the larger trophic chambers at the left side of this figure, a staining gradient indicated higher expression levels in basal nurse cells as compared to apical ones. In the egg chambers of these follicles, *Amvasa* transcripts only became visible as the oocytes started to increase in size (Figure 3D, E).

*In situ* hybridization experiments on queen ovarioles showed a similar picture, confirming that *Amvasa* transcript levels gradually increase as germ line cells pass from the upper to the lower germarium. Since the germaria of queen ovarioles are much longer than those of workers, we additionally observed that in the more basal parts of the germarium, *Amvasa* expression appeared to be highest in oocytes and/or the trophocytes closest to it (Figures 4A, E). This is the region where oocyte-trophocyte complexes undergo a transformation from a cluster to a comet-like arrangement (Tanaka and Hartfelder, 2004).

As the follicles segregated from the germarium (arrow in Figure 4A) *Amvasa* transcript levels were still higher in the oocytic than in the trophic chamber. However, soon thereafter this picture changed and *Amvasa* expression appeared to be switched on in trophocytes, which, like in the workers, now showed higher and gradually increasing *Amvasa* transcript levels (Figures 4A, B). In egg chambers, a marked increase in *Amvasa* mRNA was only observed as they enlarged and reached about the same size as the corresponding trophic chambers (Figure 4C). A thin stream of *Amvasa* transcripts (arrow in Figure 4C) indicated that *Amvasa* mRNA may be transported from the trophic chamber to the oocytes across the trophocytic canal. This panel also illustrates the apparent restriction of *Amvasa* transcripts to germ line cells since follicle epithelial cells did not stain with the probe.

**Figure 5:**
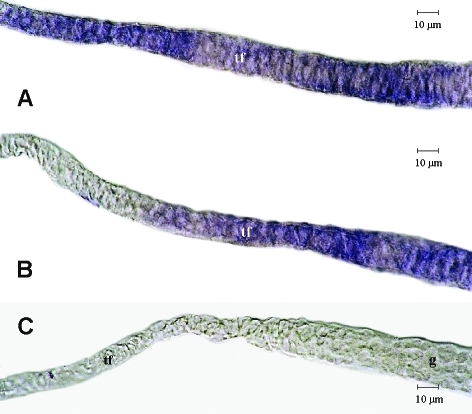
*Amvasa* mRNA detection by *in situ* hybridization on terminal filaments of ovarioles of mated queens. (A, B) Labeling for the germ line marker *Amvasa* was diffuse and variable in this region and, while indicating the presence germ cells, it did not allow distinguishing between disc-like somatic cells and rounded putative germ line stem cells. (C) Negative control with sense probe. g - germarium, tf - terminal filament.

We next investigated more closely whether *Amvasa* transcripts may be present in terminal filaments of queen ovarioles. This was of interest because previous histological studies had indicated the possible presence of germ line stem cells in niches along the extensive terminal filaments of queen ovarioles (Gutzeit *et al.*, 1993; Tanaka and Hartfelder, 2004). Even though we could detect the presence of *Amvasa* transcripts in terminal filaments (Figure 5), this staining was rather diffuse and not constant. Especially, it did not allow us to distinguish between the rounded putative germ line cells and the flattened, disc-like somatic cells of the filaments. This finding is of interest because, as described above, we also could not detect *Amvasa* transcripts in the transition zone from the terminal filament to the top of the germarium (Figure 3A), where germ cells could be clearly identified by their general histology and cell division pattern (Tanaka and Hartfelder, 2004). This finding indicates that in the honey bee, *vasa* may not be a marker appropriate for germ line stem cells, but that *vasa* apparently becomes induced in the germ line only after cystocytes separate from the niche and begin to form the large germ cell clusters, typical of the polytrophic meroistic ovary type.

### *In situ* detection of *vasa* transcripts in ovaries of the stingless bee *Melipona quadrifasciata*

**Figure 6:**
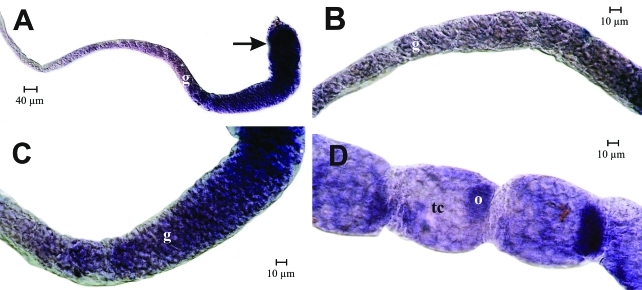
*In situ* hybridization with a honey bee *vasa* probe on ovarioles of queens of the stingless bee *Melipona quadrifasciata.* (A, B, C) *vasa* mRNA was detected in germ line cells throughout the germarium. (D) In previtellogenic follicles, *vasa* transcript levels in oocytes were higher than those seen in the corresponding trophic chambers (see also follicle marked by arrow in A). g - germarium, o - oocyte, tc - trophic chamber.

The high degree in sequence identity made it possible to use the *Amvasa* probe for *in situ* hybridization experiments on ovarioles of the stingless bee, *M. quadrifasciata*. This probe detected *vasa* transcripts throughout the entire length of the ovarioles of stingless bee queens. Like in the honey bee, *vasa* transcript levels gradually increased along the germarium accompanying oogenesis progression (Figure 6A-C). However, as the follicles separated from the germarium, the timing of *vasa* expression in the stingless bee seemed to differ from the pattern observed in the honey bee. In the honey bee, *Amvasa* transcript levels were initially higher in the oocyte, but subsequently *Amvasa* expression became strongly turned on in the trophocytes, and only later did its transcript levels gradually increase again in the oocyte. In contrast to this, we noted that *vasa* transcript levels in the stingless bee ovary were continuously higher in the oocytes, already from the start of follicle growth (Figure 6D). This difference in the localization of a presumably important component for germ line function is of interest as it may represent a molecular corollary to the morphological differences between *A. mellifera* and *M. quadrifasciata* ovarioles (Tanaka *et al.*, 2009).

## Discussion

###  Molecular analysis of the *vasa* orthologs of the honey bee and stingless bees

The predicted amino acid sequence of the *Apis mellifera* Vasa protein contained all the diagnostic motifs of DEAD-box helicases, but differed in two puzzling aspects from Vasa proteins of other insects. Most notably, the ARKF motif, which is considered a diagnostic motif of the Vasa family, had changed to IVKF (Chang *et al.*, 2002). Similar alterations in this motif had previously been reported for the parasitic wasp, *Copidosoma floridanum* (Donnell *et al.*, 2004), the red flour beetle *Tribolium castaneum* (Lorenzen *et al.*, 2005) and also for other organisms (Sagawa *et al.*, 2005; Ohashi *et al.*, 2007). The function of the ARKF domain is not clear yet, and alterations in this motif do not seem to interfere with the ATPase or helicase activities of Vasa proteins (Sagawa *et al.*, 2005; Schröder, 2006; Ohashi *et al.*, 2007).

Another difference between honey bee Vasa and its orthologs in other insects concerns the reduction in N-terminal RGG repeats. RGG motifs in human hnRNP U protein have been shown to bind RNA (Kiledjian and Dreyfuss, 1992). This function could be important in the organization of specific RNA complexes at the posterior egg pole when polar granules are formed (Hay *et al.*, 1988), even though the localization of Vasa itself to the posterior pole appears to be independent of its RNA binding capacity (Liang *et al.*, 1994).

Sequencing of *vasa* orthologs of four stingless bee species showed that the change from ARKF to an IVKF motif and the reduction in RGG repeats is shared by the two tribes Apini and Meliponini. These modifications in an otherwise highly conserved protein, thus, appear to predate the split between the two tribes of highly eusocial bees. Especially in the light of recent molecular phylogenies of the entire clade Apinae (Kawakita *et al.*, 2008; Whitfield *et al.*, 2008), they could actually represent an ancestral trait in the corbiculate bees, predating the evolution of sociality in this group. Confirming this hypothesis will, however, require sequencing of *vasa* orthologs in bumble bees (Bombini) and orchid bees (Euglossini).

The cladogram for the hymenopteran *vasa* orthologs further underlines the high degree in similarity for this gene in the two tribes of highly eusocial bees and sets them clearly apart from parasitic wasps. The fact that the two species of the genus *Melipona* cluster together is not surprising. The split between these and the two other stingless bee species (*S. postica* and *F. varia*), however is of interest. The latter two species are traditionally included within the trigonines, a grouping of several hundred stingless bee species (Camargo and Pedro, 2007). The two groups differ with respect to the mechanism of caste determination (Kerr, 1950; Hartfelder *et al.*, 2006).

###  Developmental profiling and tissue specificity of *vasa* expression in honey bees

The results of this qualitative analysis showed that *Amvasa* is expressed continuously and at apparently constant levels in the developing gonads of both queens and workers during the late larval stages (fourth and fifth instar) and during pupal development. Interestingly, the expression levels seem to be higher in queens than in workers, and this aspect certainly deserves a closer look using a quantitative RT-PCR approach. The observed caste differences might be related to the divergence in ovary development, particularly in the fifth instar which is marked by massive cell death in the ovaries of worker larvae (Hartfelder and Steinbrück, 1997; Schmidt-Capella and Hartfelder, 1998, 2002), considerably reducing the number of germ line cells.

The above described differences in amino acid sequence between honey bee Vasa and its orthologs in other insects made us ask whether this may be reflected in altered tissue specificity of *vasa* expression. Besides the clear and expected signal obtained for ovaries of adult queens and workers, we also found evidence for a low, yet clearly detectable level of *vasa* expression in fat body of honey bee queens. Since none of the other tissues showed such a signal, and since it was found in queen fat body only, we conclude that this is a relevant non-germ line expression, specific to the fat body of the reproductive caste. Knowingly, the fat body plays a major role in controlling fertility of queens and workers by its output of vitellogenin, the major yolk protein precursor. Honey bee queens are notorious for their extremely high hemolymph vitellogenin titers (Engels, 1974; Hartfelder and Engels, 1998), and the fat body is the major site of vitellogenin gene expression (Piulachs *et al.*, 2003). The finding of a *vasa* expression signal in queen fat body could, thus, provide a link between the previtellogenic and vitellogenic phases of follicle development.

Extra-gonadal expression of *vasa* is, however, neither a novel finding nor a unique one specific to honey bees. Vasa protein and/or *vasa* mRNA have, for example, already been detected in embryonic somatic cells of *Xenopus laevis* (Ikenishi and Tanaka, 2000), in polychaetes (Rebscher *et al.*, 2007), and in ectodermal epithelial cells of cnidarians (Mochizuki *et al.*, 2001). In honey bee embryos, a group of cells in the mandibular segment has been shown to express *Amvasa* (Dearden, 2006).

### *In situ* localization of *vasa* mRNA in the ovaries of highly eusocial bees

The detection of *Amvasa* mRNA throughout the germarium and in the cytoplasm of nurse cells and oocytes of follicles confirmed the expected association of *Amvasa* expression with germ line cells. Besides detecting an oocytic *vasa* mRNA signal in the lower germarium and in early follicles as they segregated from the germarium, we also found evidence for *Amvasa* expression in cells composing the terminal filaments. Divergence from the fly model of oogenesis (Bastock and St Johnston, 2008) in these two aspects raises the question as to whether honey bees may differ from *Drosophila* in pathways involving Vasa function(s). In fly oogenesis, *vasa* transcription depends on the prior localization of *oskar* mRNA to the posterior pole, and Oskar protein then remains associated with the germ line determining pole plasm (Rongo and Lehmann, 1996). In turn, Vasa protein is necessary for the localized translation of *nanos* mRNA (Gavis *et al.*, 1996) and for the localization of other gene products, including *gurken* mRNA, which is involved in the establishment of the two major oocyte and, consequently, embryonic axes (Tinker *et al.*, 1998). Since in the honey bee genome neither *gurken* nor *oskar* orthologs were found (The Honey Bee Genome Sequencing Consortium, 2006), but a clear ortholog of *nanos,* it is quite possible that embryonic axis specification and germ line determination in the honey bee follows a different route from that established for the fly. Interestingly, *gurken, oskar* and *bicoid* are also missing in the *Bombyx mori* and *Tribolium castaneum* genomes (Dearden *et al.*, 2006; Schröder, 2006) and, like in the honey bee, in neither of these species differentiated pole cells were evidenced (Nakao, 1999; Handel *et al.*, 2000). In the honey bee, germ line cells seem to be formed by an inductive event late in embryogenesis (Dearden, 2006; Fleig and Sander, 1998), as is the case in most metazoans (Extavour and Akam, 2003). Whether and how the early appearance of *Amvasa* mRNA in oocytes and trophocytes may be related to the determination of germ line cells, needs further investigation, especially as to how *vasa* expression may be connected to downstream events, such as the translation of *nanos* mRNA in honey bee eggs.

The detection by *in situ* hybridization of *Amvasa* transcripts in the terminal filament region is another clear difference to *Drosophila*, but actually did not come as a surprise. The ovarioles of *Apis mellifera* queens and workers have enormously elongated terminal filaments, and these house two cell types. Interspersed between the flattened somatic cells organized in stack-of-coins arrangements are clusters of rounded cells with large nuclei and weakly staining cytoplasm. Being mitotically active (Tanaka and Hartfelder, 2004), they show a set of characteristics normally attributed to primordial germ line cells (Mahowald, 1962; Wolf *et al.*, 1983). In contrast, terminal filaments in the *Drosophila* ovary are short and made up of disk-like somatic cells only. Apart from tethering together the ovarioles, an important function of the terminal filament is to form a cap structure that, in *Drosophila*, provides a niche environment around the 2-3 germ line stem cells (Lin and Spradling, 1993; Zhang and Kalderon, 2000; Morrison and Spradling, 2008). For sustaining high egg-laying rates, such as observed for honey bee queens, a system of 2-3 stem cells in a niche would probably be insufficient. Clusters of germ-line stem cells in elongated terminal filaments could, thus, be a solution to this problem. The rather diffuse pattern of *Amvasa* staining over the terminal filaments, however, did not permit to definitively distinguish somatic from putative germ line cells. But while this would be in accordance with epigenetic inductive events that might progressively separate germ line from somatic cells (Extavour, 2007), it is still unclear as to how such germ line cells in the terminal filament would make their way down into the germarium.

The spatial pattern of *vasa* expression observed in honey bee ovarioles was very similar to that seen in the stingless bee *Melipona quadrifasciata*. Also in this species, *vasa* transcript levels were observed to gradually increase along the apical-basal axis of the germarium, and *vasa* mRNA was also detected in early follicles and seen to accumulate in oocytes. These findings are of interest because, together with the close *vasa* sequence similarity for the two groups of highly eusocial bees, they provide a unifying theme for oogenesis in these bees, inspite of their differences in ovariole structure (Tanaka *et al.*, 2009) and in reproductive biology. This difference resides primarily in the ovarian activity of stingless bee workers which may produce both reproductive and trophic eggs (for review see Hartfelder *et al.*, 2006).

Our findings on *vasa* expression in social bees are in accordance with a recent study on *vasa* and *nanos* expression in ants (Khila and Abouheif, 2008), where Vasa protein was also detected in oocytes which were still in the germarium and in follicles that had separated from it. In ant species where workers produce two types of eggs, as do stingless bee workers, *nanos* mRNA appeared concentrated in a spot that colocalized with Vasa protein. In eggs with reproductive potential, this spot stayed associated with the posterior oocyte pole. In contrast, Vasa protein, and consequently also *nanos* mRNA, apparently failed to localize to the posterior pole in the non-viable trophic eggs. Khila and Abouheif (2008) consider that this mislocalization of two crucial posterior pole components observed in trophic eggs could represent a developmental mechanism that generates reproductive constraint in the worker caste and, thus, would represent an important factor in social evolution in the Hymenoptera. Obviously, this is an interesting hypothesis with wide ranging implications on the expression patterns of genes underlying developmental patterning mechanisms. The question is, how and to what extent these genes may have also been co-opted to generate evolutionarily stable differences in the reproductive potential of females in the caste phenotypes of other social Hymenoptera, such as wasps and bees.

## Supplementary Material

The following online material is available for this article:

Figure S1 ClustalW alignment of insect Vasa protein sequences.

Figure S2 ClustalW alignment of hymenopteran Vasa protein sequences, with *Drosophila* Vasa as outgroup.
