# Supplementary material for: Sequence and expression pattern of the germ line marker vasa in honey bees and stingless bees
Source: Genet Mol Biol. 2009 Sep 1;32(3):582–93. doi: 10.1590/S1415-47572009005000043 (PMC3036037; doi:10.1590/S1415-47572009005000043)
Supplement: Figure S1 — ClustalW alignment of insect Vasa protein sequences. [file gmb-32-3-582-suppl1.pdf]

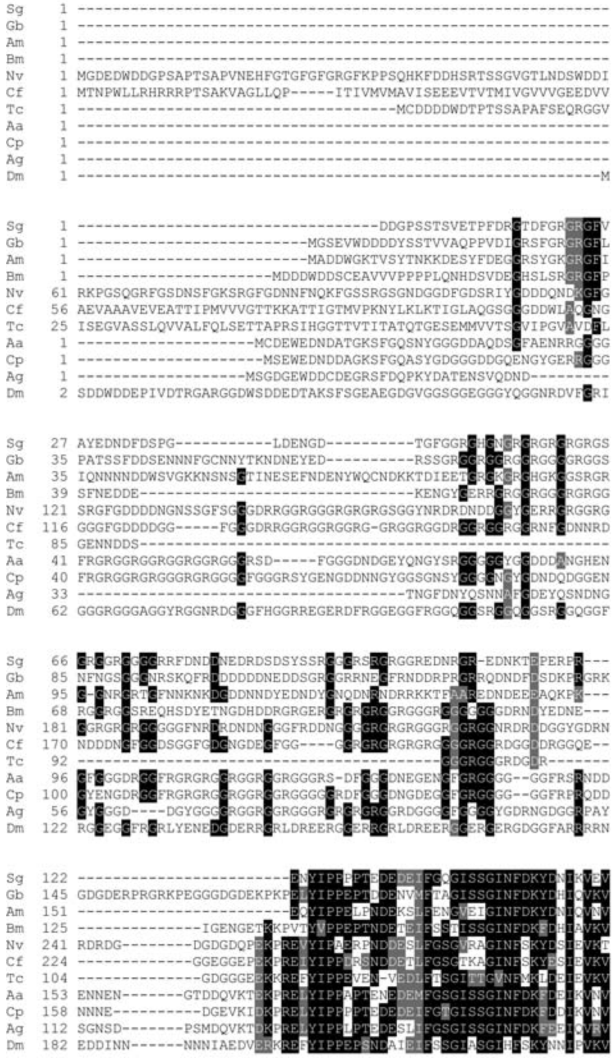

Figure S1 - ClustalW alignment of insect Vasa protein sequences.

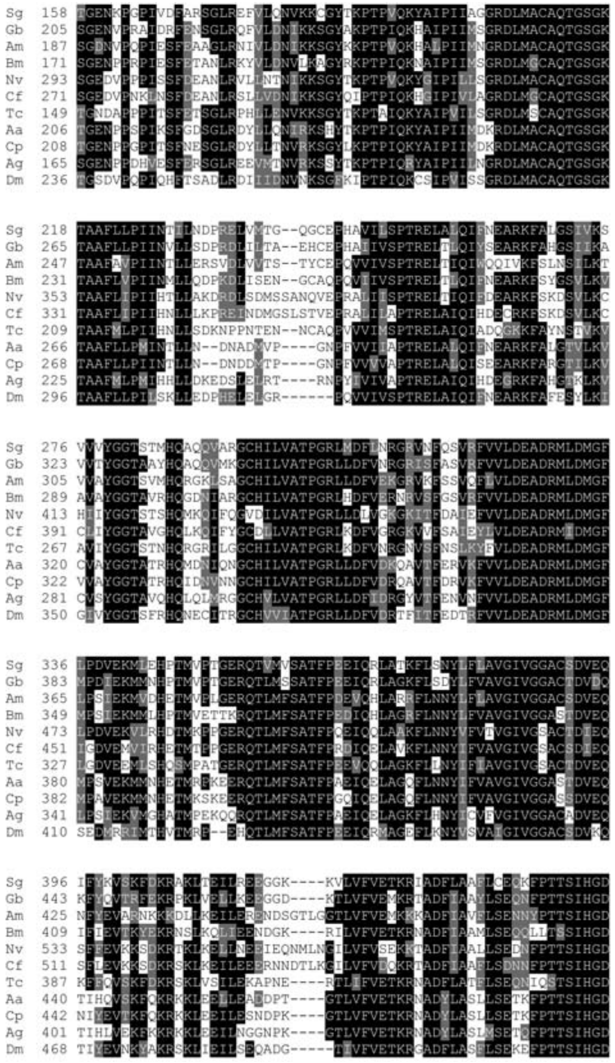

Figure S1 - (cont.)

```
Sg  452 RLQSQREEALYDFKSGRMGILVATAVAARGLDIKNVAHVINYDLPKSIDEYVHRIGRTGR
Gb  499 RMQREREEALADFKSGQMTILVATAVAARGLDIKNVAHVVNYDLPKSIDEYVHRIGRTGR
Am  485 RLQRQREEALADFKSGRMSILVATAVAARGLDIKNVSHVINYDLPKGIDEYVHRIGRTGR
Bm  465 RMQREREEALQNFKSGKHCILVATAVAARGLDIKNVDIVVNYDLPKSIDEYVHRIGRTGR
Nv  593 RLQREREEALYDFKTGKMAILVATAVAARGLDIKNVRHVINYDLPKEIDEYIHRIGRTGR
Cf  571 RLQREREEALNDFKSGRMCILVATAVAARGLDIKNVRFVINFDLPKEIDEYIHRIGRTGR
Tc  443 RYQSEREKALLDFKTGHRKVLVATGVAARGLDIKDVQHVINYDLPKSIDEYVHRIGRTGR
Aa  496 RLQREREEALRDFKSGKMFILIATSVAARGLDIKNVAHVVNYDLPKSIDDYVHRIGRTGR
Cp  498 RLQREREEALRDFKSGKMYILIATSVAARGLDIRNVAHVINYDLPKGIDDYVHRIGRTGR
Ag  457 RLQREREMALYDFKSGRMDVLIATSVAARGLDIKNVNHVVNYDLPKSIDDYVHRIGRTGR
Dm  523 RLQSQREQALRDFKNGSMKVLIATSVASRGLDIKNIKHVINYDMPSKIDDYVHRIGRTGC

Sg  512 VGNRGRATSFYDPDVDAPIARDLVKILQQANQNVPSFLESDAKG-----GVSAYRGGQFG
Gb  559 VGNRGKATSFYDPEADAPIARDLVKILNQAEQPVPEWLEQESGGSGGG-GFARGGRSGFG
Am  545 VGNRGRATSFFDPEEDAPLRGDLVRILKQANQSVPDWMMGGNANRN---FMPGRGISKFG
Bm  525 VGNRGKAVSFYDSDQDLALVADLSKILRQADQSVPDFLKG-------G-GTATFKGNKYG
Nv  653 VGNKGKATSFFDPRYDEKLQGDLVRVLTQAGQEVPDWLASGAGESNYD-GPSDFGGQDIR
Cf  631 VGNKGKAVSFYDESIDNRIQPDLVKILEMANQPIPEWLASGAGYSNLG--PSDFGAEDIR
Tc  503 VGNKGKATSFFDEDQDRNLASDLAKILSQAKQEIPEWLGS---KSYGG-SADQFGGRDIR
Aa  556 VGNKGKATSFYDMEADAAIAPDLVKILTQAGQQVPDFLEGLSTGGGSFGGGSQFGGRDIR
Cp  558 VGNKGRATSFFDMENDSAIAGDLVKILTQAGQQVPDFLQGMSGGGGSYGGPSQFGARDIR
Ag  517 VGNKGRATSFYDPEADRAMASDLVKILTQAGQSVPDFLKDAG-GSGSYMGSSQFGGKDIR
Dm  583 VGNNGRATSFFDPEKDRAIAADLVKILEGSGQTVPDFLRTCGAGGDGGYSNQNFGGVDVR

Sg  567 GSDIRNFEPENLAGPPEPEEPW-----------
Gb  618 GKDVRKFGQESSGASGGAGDYGQPQCMEEDEVW
Am  602 GEDVRESEAFAEEYTEEAYSVAQEPEEEW----
Bm  577 GSDVRNFNNANTAVVDQGQEPEEEW--------
Nv  712 NFDNAHTSGGGTKPVEEDDEW------------
Cf  689 DFPNSSGAQG-TLPVENDDAW------------
Tc  559 GDDFGRGQDFSNAAAVQEEEVW-----------
Aa  616 S-RDTGGSRVDAQPKQMEPEEDWD---------
Cp  618 GGRDAEGSRMDAQPSALEPDEEWN---------
Ag  576 D---SYGSRVDAQPVALEPEEEWE---------
Dm  643 GRGNYVGDATNVEEEEQWD--------------
```

Figure S1 - (cont.)
