# Supplementary material for: Sequence and expression pattern of the germ line marker vasa in honey bees and stingless bees
Source: Genet Mol Biol. 2009 Sep 1;32(3):582–93. doi: 10.1590/S1415-47572009005000043 (PMC3036037; doi:10.1590/S1415-47572009005000043)
Supplement: Figure S2 — ClustalW alignment of hymenopteran Vasa protein sequences, with Drosophila Vasa as outgroup. [file gmb-32-3-582-suppl2.pdf]

```
Nv      MGDEDWDDGPSAPTSAPVNEHFGTGFGFGRGFKPPSQHKFDDHSRTSSGVGTLNDSWDDIRKPGSQGRFGSDNSFGKSRGFGDNNFNQKFGSSRGSGNDGGDFGDSRIYGDDDQNDKGFG
Cf      MTNPWLLRHRRRPTSAKVAGLLQP-----ITIVMVMAVISEEEVTVTMIVGVVVGEEDVVAEVAAAVEVEATTIPMVVVGTTKKATTIGTMVPKNYLKLKTIGLAQGSGGGDDWLAQGNG
Ms      ----------------------------------------------------------MADDWGKTVSYTNKKDESYFDEGGRSYGKGRGFIIQNNNNDDWSVGKKNSNSGTINESEFND
Mq      ----------------------------------------------------------MADDWGKTVSYTNKKDESYFDEGGRSYGKGRGFIIQNNNNDDWSVGKKNSNSGTINESEFND
Sp      ----------------------------------------------------------MADDWGKTVSYTNKKDESYFDEGGRSYGKGRGFIIQNNNNDDWSVGKKNSNSGTINESEFND
Fv      ----------------------------------------------------------MADDWGKTVSYTNKKDESYFDEGGRSYGKGRGFIIQNNNNDDWSVGKKNSNSGTINESEFND
Am      ----------------------------------------------------------MADDWGKTVSYTNKKDESYFDEGGRSYGKGRGFIIQNNNNDDWSVGKKNSNSGTINESEFND
Dm      -MSDDWDDEPIVDTRG----------------------------------------ARGGDWSDDEDTAKSFSGEAEGDGVGGSGGEGGGYQGGNRDVFGRIGGGRGGGAGGYRGGNRDG
                                                                                .          .          .              ..     :  .

Nv      SRGFGDDDDNGNSSGFSGGGDRRGGRGGGRGRGRGSGGYNRDRDNDDGGYGERRGRGGRGGGRGRGRGGGGGFNRDRDNDNGGGFRDDNGGGGRGRGRGGGRGGRGGNRDRDDGGYGDRN
Cf      GGGFGDDDDGG-----FGGGDRRGGRGGRGGRG-GRGGRGGDRGGRGGRGGRNFGDNNRDNDDDNGFGGDSGGFGDGNGDEGFGG----GGRGRGRGRGRGGGGRGGRDGGDDRGGQE--
Ms      ENYWQCNDKKTDIEETGRGKGRGHGKGGSRGRGGDRGRTGFNNKNKDGDDNNDYEDNDYQ--DNRNDRRKKTFAARENNDE---------------------------------------
Mq      ENYWQCNDKKTDIEETGRGKGRGHGKGGSRGRGGDRGRTGFNNKNKDGDDNNDYEDNDYQ--DNRNDRRKKTFAARENNDE---------------------------------------
Sp      ENYWQCNDKKTDIEETGRGKGRGHGKGGSRGRGGNRGRTGFNNKNKDGDDNNDYEDNDYQ--DNRNDRRKKTFAAREDNDE---------------------------------------
Fv      ENYWQCNDKKTDIEETGRGKGRGHGKGGSRGRGGNRGRTGFNNKNKDGDDNNDYEDNDYQ--DNRNDRRKKTFAAREDNDE---------------------------------------
Am      ENYWQCNDKKTDIEETGRGKGRGHGKGGSRGRGGNRGRTGFNNKNKDGDDNNDYEDNDYGNQDNRNDRRKKTFAAREDNDE---------------------------------------
Dm      GGFHGGRREGERDFRGGEGGFRGGQGGSRGGQGGSRGGQGGFRGGEGGFRGRLYENEDGDERRGRLDREERGGERRGRLDREERG----------------GERGERGDGGFARRRRNED
                .         *  *    *.  *:* . *  .  .  . .*  ..     .      ..              *.

Nv      RDRDGDGDGDQPEKPREVYIPAERPNDDESLFGSGVRAGINFSKYDSIEVKTSGEDVPPPISSFDEANLRVLLNTNIKKSGYTKPTPVQKYGIPILLSGRDLMACAQTGSGKTAAFLIPI
Cf      -----GGEGGEPEKPREIYIPPDRSNDDETLFGSGTKAGINFSKYESIEVKVSGEDVPNKLNSFDEANLRSLLVDNIKKSGYQIPTPIQKHGIPIVLAGRDLMGCAQTGSGKTAAFLIPI
Ms      ---------EEAQKPKEQYIPPELPNDEKSLFENGVEIGINFDKYDNIQVNVSGDNVPQPIESFEAAGLRNIVLDNIKKSGYKKPTPVQKHALPIIMNGRDLMACAQTGSGKTAAFAVPI
Mq      ---------EEAQKPKEQYIPPELPNDEKSLFENGVEIGINFDKYDNIQVNVSGDNVPQPIESFEAAGLRNIVLDNIKKSGYKKPTPVQKHALPIIMNGRDLMACAQTGSGKTAAFAVPI
Sp      ---------GEAQKPKEQYIPPELPNDEKSLFENGVEIGINFDKYDNIQVNVSGDNVPQPIESFEAAGLRNIVLDNIKKSGYKKPTPVQKHALPIIMNGRDLMACAQTGSGKTAAFAVPI
Fv      ---------GEAQKPKEQYIPPELPNDEKSLFENGVEIGINFDKYDNIQVNVSGDNVPQPIESFEAAGLRNIVLDNIKKSGYKKPTPVQKHALPIIMNGRDLMACAQTGSGKTAAFAVPI
Am      ---------EEAQKPKEQYIPPELPNDEKSLFENGVEIGINFDKYDNIQVNVSGDNVPQPIESFEAAGLRNIVLDNIKKSGYKKPTPVQKHALPIIMNGRDLMACAQTGSGKTAAFAVPI
Dm      DINNNNNIAEDVERKREFYIPPEPSNDAIEIFSSGIASGIHFSKYNNIPVKVTGSDVPQPIQHFTSADLRDIIIDNVNKSGFKIPTPIQKCSIPVISSGRDLMACAQTGSGKTAAFLLPI
                  : :: :* ***.: .**   :* .*   **:*.**:.* *:.:*.:**  :. *  *.** ::  *::***:  ***:** .:*::  *****.************ :**

Nv      IHTLLAKDRDLSDMSSANQVEPRALIISPTRELTIQIFDEARKFSKDSVLKCHIIYGGTSTSHQMKQIFQGVDILVATPGRLLDLVGKGKITFDAIEFVVLDEADRMLDMGFLPDVEKVL
Cf      IHNLLLKPREINDMGSLSTVEPRALILAPTRELAIQIHDECRKFSKDSVLKCCLIYGGTAVGHQLKQIFYGCDLLVATPGRLKDFVGRGKVVFSAIEYLVLDEADRMIDMGFIGDVEMVI
Ms      INTLLER--SVDLVVTSTYCEPQVVIVSPTRELTIQIWQQIVKFSLNSILKTVVAYGGTSVMHQRGKLSAGCHILVATPGRLLDFVEKGRVKFSSVQFLVLDEADRMLDMGFLPSIEKMV
Mq      INTLLER--SVDLVVTSTYCEPQVVIVSPTRELTIQIWQQIVKFSLNSILKTVVAYGGTSVMHQRGKLSAGCHILVATPGRLLDFVEKGRVKFSSVQFLVLDEADRMLDMGFLPSIEKMV
Sp      INTLLER--SVDLVVTSTYCEPQVVIVSPTRELTIQIWQQIVKFSLNSILKTVVAYGGTSVMHQRGKLSAGCHILVATPGRLLDFVEKGRVKFSSVQFLVLDEADRMLDMGFLPSIEKMV
Fv      INTLLER--SVDLVVTSTYCEPQVVIVSPTRELTIQIWQQIVKFSLNSILKTVVAYGGTSVMHQRGKLSAGCHILVATPGRLLDFVEKGRVKFSSVQFLVLDEADRMLDMGFLPSIEKMV
Am      INTLLER--SVDLVVTSTYCEPQVVIVSPTRELTIQIWQQIVKFSLNSILKTVVAYGGTSVMHQRGKLSAGCHILVATPGRLLDFVEKGRVKFSSVQFLVLDEADRMLDMGFLPSIEKMV
Dm      LSKLLEDP------HELELGRPQVVIVSPTRELAIQIFNEARKFAFESYLKIGIVYGGTSFRHQNECITRGCHVVIATPGRLLDFVDRTFITFEDTRFVVLDEADRMLDMGFSEDMRRIM
        : .**               .*:.:*::*****:*** ::  **: :* **  : ****:  **   :  * .:::****** *:* :  : *.  .::********:****  .:. ::

Nv      RHDTMKPPGERQTLMFSATFPQEIQQLAAKFLNNYVFVTVGIVGSACTDIEQSFFEVKKSDKRTKLKELLNEEIEQNMLNGILVFVSEKKTADFIAALLSEDNFPTTSIHGDRLQREREE
Cf      RHETMTPPGERQTLMFSATFPRDIQELAVKFLNNYIFVAVGIVGSACSDIEQSFLEVKKSDKRSKLKEILEEERNNDTLKGILVFVDQKRTADFIAAFLSDNNFPTTSIHGDRLQREREE
Ms      DHETMVPLGERQTLMFSATFPDEVHHLARRFLNNYLFLAVGIVGGARSDVEQNFCEVARNKKKDLLKEILERENDSGTLGGTLVFVEMKKKADFIAVFLSENNYPTTSIHGDRLQRQREE
Mq      DHETMVPLGERQTLMFSATFPDEVHHLARRFLNNYLFLAVGIVGGARSDVEQNFCEVARNKKKDLLKEILERENDSGTLGGTLVFVEMKKKADFIAVFLSENNYPTTSIHGDRLQRQREE
Sp      DHETMVPLGERQTLMFSATFPDEVQHLARRFLNNYLFLAVGIVGGACSDVGRNFYEVARNKKKDLLKEILERENDSGTLGGTLVFVEMKKKADFIAVFLSENNYPTTSIHGDRLQRQREE
Fv      DHETMVPLGERQTLMFSATFPDEVQHLARRFLNNYLFLAVGIVGGACSDVGRNFYEVARNKKKDLLKEILERENDSGTLGGTLVFVEMKKKADFIAVFLSENNYPTTSIHGDRLQRQREE
Am      DHETMVPLGERQTLMFSATFPDEVQHLARRFLNNYLFLAVGIVGGACSDVEQNFYEVARNKKKDLLKEILERENDSGTLGGTLVFVEMKKKADFIAVFLSENNYPTTSIHGDRLQRQREE
Dm      THVTMRP--EHQTLMFSATFPEEIQRMAGEFLKNYVSVAIGIVGGACSDVKQTIYEVNKYAKRSKLIEILSEQADG-----TIVFVETKRGADFLASFLSEKEFPTTSIHGDRLQSQREQ
         * ** *  *:*********** :::.:* .**:**: :::****.* :*: :.: ** :  *:  * *:*..: :        :***. *: ***:* :**:.::*********** :**:

Nv      ALYDFKTGKMAILVATAVAARGLDIKNVRHVINYDLPKEIDEYIHRIGRTGRVGNKGKATSFFDPRYDEKLQGDLVRVLTQAGQEVPDWLASGAGESNYDG---PSDFGGQDIRNFDNAH
Cf      ALNDFKSGRMCILVATAVAARGLDIKNVRFVINFDLPKEIDEYIHRIGRTGRVGNKGKAVSFYDESIDNRIQPDLVKILEMANQPIPEWLASGAGYSNLG----PSDFGAEDIRDFPNSS
Ms      ALADFKSGRMSILVATAVAARGLDIKNVSHVINYDLPKGIDEYVHRIGRTGRVGNRGRATSFFDPEEDAPLRGDLVRILKQANQSVPDWMMGGNANRNFMPGGGISKFGGEDVRESEAFA
Mq      ALADFKSGRMSILVATAVAARGLDIKNVSHVINYDLPKGIDEYVHRIGRTGRVGNRGRATSFFDPEEDAPLRGDLVRILKQANQSVPDWMMGGNANRNFMPGGGISKFGGEDVRESEAFA
Sp      ALADFKSGRMSILVATAVAARGLDIKNVSHVINYDLPKGIDEYVHRIGRTGRVGNRGRATSFFDPEEDAPLRGDLVRILKQANQSVPDWMMGGNANRNFMPGRGISKFGGEDVRESEAFA
Fv      ALADFKSGRMSILVATAVAARGLDIKNVSHVINYDLPKGIDEYVHRIGRTGRVGNRGRATSFFDPEEDAPLRGDLVRILKQANQSVPDWMMGGNANRNFMPGRGISKFGGEDVRESEAFA
Am      ALADFKSGRMSILVATAVAARGLDIKNVSHVINYDLPKGIDEYVHRIGRTGRVGNRGRATSFFDPEEDAPLRGDLVRILKQANQSVPDWMMGGNANRNFMPGRGISKFGGEDVRESEAFA
Dm      ALRDFKNGSMKVLIATSVASRGLDIKNIKHVINYDMPSKIDDYVHRIGRTGCVGNNGRATSFFDPEKDRAIAADLVKILEGSGQTVPDFLRTCG--AGGDGGYSNQNFGGVDVRGRGNYV
        ** ***.* * :*:**:**:*******: .***:*:*. **:*:******* ***.*:*.**:*   *  :  ***::*  :.* :*:::        .        ..**. *:*

Nv      TSGGGTKPVEEDDEW---
Cf      GAQG-TLPVENDDAW---
Ms      EEYTEEAYSVAQEP----
Mq      EEYTEEAYSVAQEP----
Sp      EEYTEETYSVAQEP----
Fv      EEYTEETYSVAQEP----
Am      EEYTEEAYSVAQEPEEEW
Dm      GDATNVEEEEQWD-----
                    :
```

Figure S2 - ClustalW alignment of hymenopteran Vasa protein sequences, with *Drosophila* Vasa as outgroup.
